# Parental Quality of Life and Involvement in Intervention for Children or Adolescents with Autism Spectrum Disorders: A Systematic Review

**DOI:** 10.3390/jpm11090894

**Published:** 2021-09-08

**Authors:** Alessandro Musetti, Tommaso Manari, Barbara Dioni, Cinzia Raffin, Giulia Bravo, Rachele Mariani, Gianluca Esposito, Dagmara Dimitriou, Giuseppe Plazzi, Christian Franceschini, Paola Corsano

**Affiliations:** 1 Department of Humanities, Social Sciences and Cultural Industries, University of Parma, Borgo Carissimi 10, 43121 Parma, Italy; tommaso.manari@unipr.it (T.M.); barbara.dioni@unipr.it (B.D.); paola.corsano@unipr.it (P.C.); 2 Fondazione Bambini e Autismo Onlus, 33170 Pordenone, Italy; c.raffin@bambinieautismo.org (C.R.); g.bravo@bambinieautismo.org (G.B.); 3 Department of Dynamic and Clinical Psychology, Sapienza University of Rome, 00185 Rome, Italy; rachele.mariani@uniroma1.it; 4 Social and Affective Neuroscience Lab, Psychology Program-SSS, Nanyang Technological University, Singapore 639818, Singapore; gianluca.esposito@unitn.it; 5 Lee Kong Chian School of Medicine, Nanyang Technological University, Singapore 636921, Singapore; 6 Affiliative Behaviour and Physiology Lab, Department of Psychology and Cognitive Science, University Trento, 38068 Rovereto, Italy; 7 Sleep Education and Research Laboratory, Department of Psychology and Human Development, UCL-Institute of Education, London WC1H 0AA, UK; d.dimitriou@ucl.ac.uk; 8 The National Institute for Stress, Anxiety, Depression and Behavioural Change (NISAD), 252 21 Helsingborg, Sweden; 9 Department of Biomedical, Metabolic and Neural Sciences, University of Modena and Reggio Emilia, 41125 Modena, Italy; giuseppe.plazzi@unimore.it; 10 IRCCS Institute of Neurological Sciences of Bologna (ISNB), 40139 Bologna, Italy; 11 Department of Medicine and Surgery, University of Parma, 43121 Parma, Italy; Christian.franceschini@unipr.it

**Keywords:** autism spectrum disorder, quality of life, parents, intervention, systematic review

## Abstract

Previous research has examined several parental, child-related, and contextual factors associated with parental quality of life (QoL) among parents with a child or an adolescent with autism spectrum disorders (ASD); however, no systematic review has examined the relationship between parental QoL and parental involvement in intervention. To fill this gap, a systematic review was conducted using four electronic databases and checked reference lists of retrieved studies. Records were included in the systematic review if they presented original data, assessed parental QoL, and involvement in intervention for children or adolescents with ASD, were published in peer-reviewed journals between 2000 and 2020, and were written in English. Among the 96 screened full-texts, 17 articles met the eligibility criteria. The selected studies included over 2000 parents of children or adolescents with ASD. Three categories of parental involvement (i.e., none, indirect, direct) were identified, which varied across studies, although most had direct parental involvement. The results from this review show that increased parental involvement in the intervention for children or adolescents with ASD may be one way to promote their QoL. However, further research specifically focused on parental involvement during the intervention for children and adolescents with ASD is warranted.

## 1. Introduction

Autism spectrum disorder (ASD) is a pervasive neurodevelopmental disorder characterised by persistent atypicalities in social communication and social interactions across different domains, together with restricted, repetitive, stereotyped patterns of behaviour, interest, or activities [1]. Although there is large variability in the expression of ASD symptoms, many individuals with ASD need lifelong assistance in daily life that is usually provided by family members, especially parents [2]. Therefore, the presence of a family member with ASD features such as challenging behaviours [3,4] or sleep problems [5,6] can significantly affect and challenge the entire family system [7], with a mostly negative impact on the quality of life (QoL) and on the relationship quality of closer family members such as siblings [8,9] and parents [10,11].

Although a lot of research has been conducted to examine the relationship between the characteristics of offspring with ASD, distress and psychological difficulties of family members [12,13], less attention has been paid to the impact of the characteristics of the intervention on family functioning [14,15]. Moreover, most of the research on interventions for ASD has so far been focused on child outcomes, disregarding the impact on parents [14]. This is surprising, given the increasing parental involvement in activities of children and adolescents with ASD [16,17] including the intervention process [18]. In many cases, parental involvement in intervention covers a broad range of activities, from parent training and homework routines, even participation in intervention design and implementation [19]. Therefore, taking into account the family outcomes, it would be possible to gain a more comprehensive view of the effectiveness of an intervention and, in turn, better shape intervention design and implementation [14,20,21]. Most early research in this field unilaterally focused on the negative outcomes (e.g., parental stress) of having a child with ASD [22]. However, a growing number of studies have investigated parental QoL in an endeavour to attempt to capture the variability of parental adaptation and to more deeply comprehend difficulties faced by these parents [23,24]. This interesting broadening of perspective highlights how some parents can cope with the stress resulting from caring for their children with ASD, and in turn, learn and improve their competencies in the process [25,26]. For example, when mothers are able to gain emotional resolution on their child’s diagnosis of ASD, they tend to develop a more supportive parenting style [27]. QoL is a multidimensional and wide-ranging construct, characterized by the individual’s emotional, physical, and financial well-being, interpersonal relationships, goals, expectations and concerns, and their interactions with salient features of the environment [28,29]. According to the World Health Organization (WHO) Quality of Life Assessment Group [30], QoL is defined as an “individual’s perception of their position in life in the context of the culture and value systems in which they live, and in relation to their goals, expectations, standards, and concerns” (p. 11). Up to date, only one study [24] has systematically reviewed published literature about the parental QoL of children and adolescents with ASD. Thus far, several variables have been found to be associated with lower QoL among parents of children and adolescents with ASD including parental characteristics (e.g., being a mother, parental mental health problems, maladaptive parental coping strategies, and low parental self-efficacy), child characteristics (e.g., child behavioural problems, child emotional problems, ASD severity, and child’s age), and contextual factors (e.g., low employment status, low household income, low availability of social and professional support, and lack of participation in health promoting activities). However, no systematic review exists that summarizes the currently available evidence on the relationship between parental QoL and parental involvement in intervention for their children or adolescents with ASD. This constitutes a relevant gap in the literature considering the recent increased emphasis on family-focused versus professional-driven interventions [14,31], which the present study intends to fill. Specifically, a previous systematic review on parental QoL of children of ASD by Vasilopoulou et al. [24] revealed a need for greater focus on parents to provide tailored intervention for this population. The current review aimed to identify and discuss the role of parental involvement in intervention in relation to QoL among the parents of children or adolescents with ASD.

## 2. Methods

### 2.1. Protocol and Registration

The present systematic review adhered to the Preferred Reporting Items for Systematic Reviews and Meta-Analyses (PRISMA) statement [32]. The protocol was registered at the International Prospective Register of Systematic Reviews (PROSPERO) data repository in February 2021 (registration code: CRD42021230103).

### 2.2. Study Selection

The selection process identified the following eligibility criteria. Inclusion criteria (IC): (IC1) studies had to be focused on parents of children and adolescents (i.e., <21 years old) with a formal diagnosis of ASD; (IC2) they had to contain quantitative, qualitative, or mixed methods approaches; and (IC3) be published between 2000 and 2020 in (IC4) peer-reviewed journals and written in English. We rejected articles that met one of the following excluding criteria (EX): (EX1) case reports, commentaries, editorials, meeting abstracts were not considered; (EX2) other review articles were consulted but not included finally and to narrow our search procedure, and we excluded (EX3) studies that dealt with the quality of life of parents of children with disabilities different from ASD.

A systematic search was carried out in January 2021, in four online databases: Scopus, Web of Science, PubMed, and PsycINFO. To reduce the risk of methodological biases, no filters were applied in the preliminary searches. We combined the selected keywords with the Boolean operators AND/OR in the following order: (“parent*”) AND (“ASD”) OR (“autis*”) AND (“quality”) AND (“life”) AND (“intervent*”), in Titles, Abstracts, and Keywords.

All references collected through database searches were exported to the systematic reviews web application Rayyan (https://rayyan.qcri.org/ (accessed on 1 August 2021)). Duplicate records were ruled out, then the titles and abstracts of all remaining articles were independently screened. The use of multiple reviewers may reduce the risk of rejecting relevant reports, as noted by Edwards and colleagues [33]. Results were then compared and in the case of disagreement, the conflicting choices were inspected and discussed further until a consensus was reached. The second step included the screening of the full-texts of the chosen studies, in order to select those where the quality of life was a specified outcome. Reference lists of relevant studies and reviews were examined for additional pertinent records. The articles deemed eligible by all reviewers and that met all the inclusion criteria were included for the final screening, and those that met at least one exclusion criteria were formally excluded with reasons.

### 2.3. Quality Assessment

The critical assessment of each paper was conducted with AXIS, a quality assessment tool for observational and cross-sectional studies [34]. It comprises a checklist of 20 items to evaluate the overall study design as well as the risk of bias. The items were scored as follows: Yes = 1, No and Don’t know = 0, and resulted in a final score that ranged from 0 to 20, with higher scores indicating a higher assessed quality. Adopting the classification used in other literature reviews (e.g., [35]), we further distinguished three quality ratings: low quality (0–7 points), moderate quality (8–14 points), and high quality (15–20 points). The qualitative assessment outcome is discussed in the Results section.

## 3. Results

### 3.1. Study Selection

**Figure 1:**
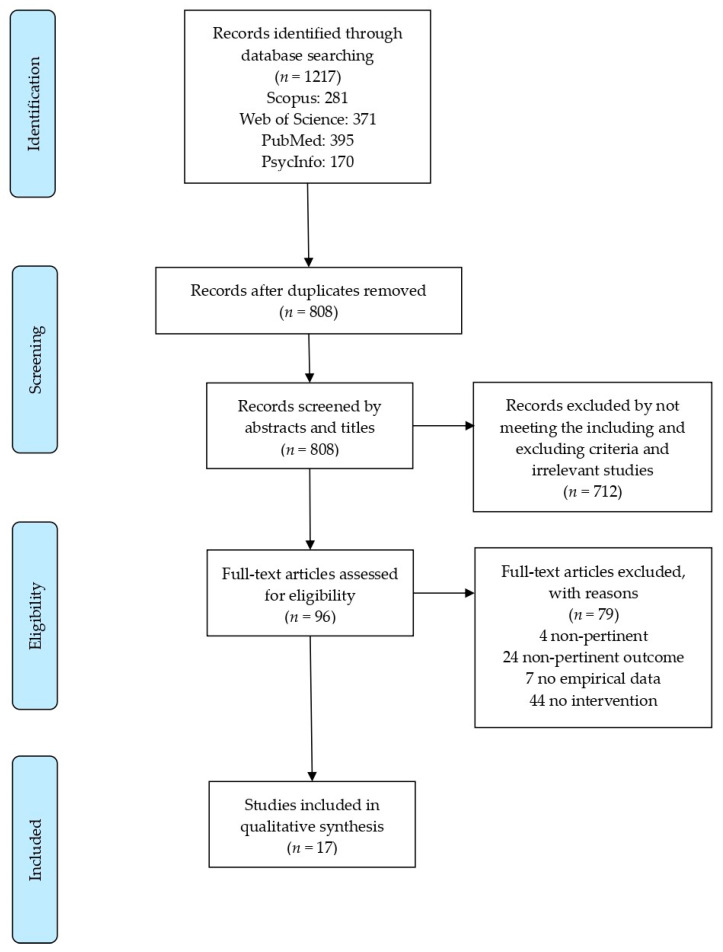
Flow diagram of the search strategy. Modified from the Preferred Reporting Items for Systematic reviews and Meta-Analyses statement flow diagram [32].

The systematic search was first performed in four online databases: Scopus, Web of Science, PubMed, and PsycINFO, and yielded a total of 1217 records. Duplicate articles were removed with the reference manager Mendeley desktop (https://www.mendeley.com/ (accessed on 1 August 2021)) and resulted in 808 unique references, which were imported to the systematic reviews web application Rayyan (https://rayyan.qcri.org/ (accessed on 1 August 2021)). Titles and abstracts were then independently screened for potentially relevant papers, resulting in 96 eligible references and 712 excluded because they did not meet the predefined inclusion criteria. As the last step, 79 full-texts were excluded with reasons (e.g., non-pertinent, non-pertinent outcome, no empirical data, or without intervention for children with ASD) and 17 articles were found to be appropriate for the qualitative synthesis. The four steps defined in the PRISMA procedure (i.e., Identification, Screening, Eligibility, and Inclusion) are represented in Figure 1 and the excluded articles are listed in Supplementary Table S1.

### 3.2. Study Characteristics

A summary of the information extracted from each study is presented in Table 1. The present systematic review examined 17 studies that were published between 2011 and 2020 in peer-reviewed journals that focused on the intrafamilial QoL of parents with a child or an adolescent affected by ASD. The adopted methodological designs were mixed and included cross-sectional designs, qualitative and observational studies, randomized controlled trials, and a validation study.

The reviewed studies involved a total of 1965 children and adolescents and 2040 parents or primary caregivers (partial information was available from three studies). The age of the offspring ranged from 2 to 20 years old (*M* = 7.43, *SD* = 3.22), and 74.48% of the involved participants were female (mothers or female caregivers; partial information was available from three studies).

In relation to the geographical distribution (i.e., where the study was conducted and the data was collected), the 17 studies were carried out in Europe (*n* = 8, United Kingdom, France, Sweden), North America (*n* = 2, United States, Canada), Asia (*n* = 2, China, Taiwan), and Australia (*n* = 5).

The present systematic review was focused on parenting education programmes and interventions. Similarly to previous studies (e.g., [36]), we distinguished between three levels of parental involvement: none (i.e., parents are not involved in intervention design and implementation), indirect (i.e., parents are involved in secondary forms of interventions such as parent training or homework routines), and direct (i.e., parents are actively involved in primary intervention development and implementation).

Accordingly, we identified a heterogeneous number of studies that included no (*n* = 2), indirect (*n* = 3), or direct (*n* = 12) levels of parental involvement. The interventions were performed in clinical settings (*n* = 7), at home (*n* = 4), or a combination of the two (i.e., mixed settings, n = 3; information not available for three records).

The construct dimensions related to the parental QoL were assessed with multiple instruments (see Table 2) including the Family Quality of Life Scale [37], the Parenting Stress Index [38], and the WHOQOL-BREF [28]. Since different studies implemented qualitative and observational approaches, semi-structured interviews, questionnaires, and ad-hoc rating scales were also used.

**Table 1:** Characteristics of the reviewed studies (*n* = 17).

Authors (Year)	Location	Study Design	Sample Characteristics *N* Age = *M* (*SD*)	Parental Involvement	Intervention	Setting	Length/Frequency	Instruments	Relevant Findings
Aandersson et al. (2017) [39]	Sweden	Qualitative study	56 Parents of Children with ASD (*n* = 56 Children; *M*_age_ ≈ 9 Years, Female = 23.21%)	Indirect	Early Intervention, ABA	Clinics	2 Years/25 h/Week	Semi-Structured Questionnaire	Thematic Content Analysis Too much responsivity and lack of knowledge about intervention methods. Perceived support as unequal, uncoordinated, and with variations. Unequal treatments depending on socioeconomic status. lack of individualization of interventions.
Baghdadli et al. (2014) [10]	France	Cross-Sectional	152 Mothers (92.12%) and 13 Fathers of Adolescents with ASD (*n* = 152 Adolescents; *M*_age_ = 15 Years; *SD* = 1.6, Female = 17.8%)	None	Mixed	Clinics	NA/31.3 5 h/week	Par-DD-QoL	Polytomic Logistic Regression Analysis A higher number of hours of specialized intervention is associated with lower parental emotional QoL (ORa = 2.69, 95% CI = 1.1–5.9, *p* = 0.04).
Derguy et al. (2018) [40]	France	Cross-Sectional	115 Parents (Female = 63.5%) of Children with ASD (*n* = 78 Children; *M*_age_ = 6.3, *SD* = 2.3, Female = 23.1%)	None	Psycho-Educational Intervention (74% of the Sample)	NA	NA	WHOQOL-BREF	Hierarchical Regression Analyses Parents showed better QoL whether their child received psychoeducation intervention (β = 0.25, *p* = 0.010).
Due et al. (2018) [41]	Australia	Mixed-Method Study	27 Parents (Female = 48.14%) of Children with ASD (*n* = 27 Children; *M*_age_ = 5, *SD* = 2.0, Female = 18.52%)	Direct	Early intervention	Clinics	NA	Quality of Life in Autism Questionnaire; Semi-Structured Interviews	Thematic Content Analysis Parental direct involvement in the intervention increased several aspects of their QoL (e.g., sense of competence and confidence as parents, community participation).
Hwang et al. (2015) [42]	Taiwan	Pre-Post Design	6 Mother-Child dyads (Children with ASD; Age Range = 8–15 Years, Female = 20%)	Direct	Parent-Mediated Home-Based Training	Home	12 Months	Family Quality of Life (FQoL)	Paired Sample Wilcoxon Signed Rank Test The parent-mediated home-based training was associated with marginally significant increase in family quality of life.
Ji et al. (2014) [43]	China	Quasi-Experimental Design	22 Caregivers (Female = 90.9%) of Children with ASD (Intervention Group, *n* = 22 Children with ADS; *M*_age_ = 4.93, *SD* = 2.03, Female = 18.2%) and 20 Caregivers (Female = 90.0%) of Children with ASD (control group, *n* = 20 Children with ASD, *M*_age_ = 5.65, *SD* = 1.74, Female = 15%)	Direct	Parent Education Program	Clinics	8 Weeks	Caregiver Burden Index (CBI)	Independent-Samples *t*-test Parents’ mental HRQOL significantly improved after the intervention (*t* = −2.138; *p* = 0.039). Parents’ physical HRQOL did not improve after the intervention (*t* = −1463; *p* = 0.151).
Jones et al. (2017) [44]	Canada	Cross-Sectional	151 Caregivers (Female = 78.95%) of children with ASD (*n* = 151 Children with ASD; *M*_age_ = 7.3, SD = 3.9, Female = 23.84%)	Indirect	None/Intensive Behavioural Intervention, Intervention from a Speech and Language Pathologist, Occupational Therapy, Physiotherapy Services	NA	NA	Family Quality of Life Survey (FQOLS)	Correlation Analyses Time on waiting list was not significatively associated with family QoL.
Leadbitter et al. (2018) [45]	United Kingdom	Pre-Trial Research Design	152 Parents of Children with ASD (*n* = 152 Children with ASD, *M*_age_ = 45 Months, Female = 9.21%)	Direct	Parent-Mediated Video-Aided Pre-School Communication-Focused Intervention	Clinics	13 Months	Autism Family Experience Questionnaire (AFEQ)	Effect Estimation Analysis AFEQ total score improved significantly after the treatment and at the 6-year follow-up.
Leadbitter et al. (2020) [46]	United Kingdom	Qualitative Study	18 Parents (Female = 66.67%) of Children with ASD (*n* = 12 Children with ASD, *M*_age_ = 44.42 Months, *SD* = 7.04, Female = 8.33%)	Direct	Paediatric Autism Communication Therapy	NA	12 Months	Semi-Structured Interview	Thematic Content Analysis Post-intervention improved family wellbeing.
Mathew et al. (2019) [47]	Australia	Cross-Sectional	161 Parents (Female = 90.7%) of Children with ASD (*n* = 117 Children with ASD, *M*_age_ = 4.13, *SD* = 0.53, Female = 17.9%)	Direct	Early Intervention using the Early Start Denver Model	Clinics	NA	Parenting Sense of Competence Scale (PSOC)	Independent-Samples *t*-test Significantly greater levels of depression symptoms, anxiety, and stress among mothers (who have the primary caregiver role) than among fathers of children with ASD.
McConkey (2020) [48]	United Kingdom	Validation Study	449 Parents (Female = 92.6%) of Children with ASD or with Suspected ASD (*n* = 449 Children with ASD, Age Range = 2–11 years, Female = 25.6%)	Direct	Family-Centred Intervention	Home	Median = 8 Weeks	Eight Items Questionnaire	Step-Wise, Linear Regression Parental with adequate or poor engagement in intervention showed significantly lower parental well-being than those with good engagement in intervention (β = 4.72; *SE* 1.54; *p* < 0.002).
McConkey et al. (2020) [49]	United Kingdom	Observational Study	92 Families with Children with ASD (*n* = 96 Children, *M*_age_ = 7.7, Female = 20.8%)	Direct	Post-Diagnostic Support	Home	12 Months	Two Rating Scales	Thematic Content Analysis Parents reported higher self-confidence and reduced stress within the family after the intervention.
McPhilemy & Dillenburger (2013) [50]	United Kingdom	Observational Study	15 Families with Children or Adolescents with ASD (*n* = 17 Children or Adolescents with ASD, *M*_age_= 38 Months, Age Range = 2–20 Years, Female = 11.76%)	Direct	ABA-based Home Program	Home	*M* = 48 Months	Questionnaire	Thematic Content Analysis ABA-based home program had significant positive impact on family quality of life.
Milbourn et al. (2017) [51]	Australia/Sweden	Observational/Qualitative Study	521 Caregivers (Female = 81%) with Children with ASD (*n* = 400 Children with ASD (76.78%), *M*_age_ = 9.92, *SD* = 4.17, Female = 17%)	Direct	Mixed	NA	NA	Multidimensional Questionnaire	Thematic Content Analysis Respondents indicated that early interventions improved their family life (71.3% answered “somewhat” or “definitely”). Respondents agreed that earlier access to intervention would have led to improved child’s quality of life (78.4% answered “somewhat” or “definitely”).
Moody et al. (2019) [52]	United States	Randomised Controlled Trial	67 Parents (Active Group, Female = 91.0%; Waitlist, Female = 88.2%) of Children with ASD (*n* = 33 Intervention Group, Female = 18.2%) (*n* = 67 Children with ASD, Range age 2–8 Years, Female = 14.7%)	Direct	Colorado Parent Mentoring (CPM) Program	Mixed	Six Months	Family Quality of Life Survey (FQOLS)	Mixed Modelling CPM program positively impacted several areas of family quality of life, regardless the amount of formal intervention received.
Paynter et al. (2018) [53]	Australia	Mixed Methods	26 Fathers of Children with ASD (*n* = 26 Children with ASD, Age Range 2.5–6 Years, Female = 26.4%)	Indirect	Early Intervention	Clinics	NA	Parenting Stress Index (PSI)	Thematic Content Analysis According to some fathers’ family adaptation was negatively affected by inaccessible and/or gender-biased formal supports.
Roberts et al. (2011) [54]	Australia	Randomised Controlled Trial	84 Children with ASD (*n* = 27 Home-Based Group, *n* = 29 Centre-Based Group, *n* = 28 Control, *M*_age_ = 3.5, *SD* = 0.61, Female = 9.5%)	Direct and Indirect	Early Intervention (Home-based Intervention vs. Centre-Based Intervention)	Home vs. Clinical Centres	1 Year (40 Weeks)	Family Quality of Life Survey (FQOLS), Parenting Stress Index (PSI)	Analysis of Covariance Parents of children who followed the centre-based intervention showed significant improvements in family QoL. Parents of children who followed the home-based intervention showed the least improvement in family QoL over all groups.

**Table 2:** Measurement instruments and construct dimensions listed in the included studies (*n* = 17).

	Measures of QoL	Construct Dimensions	Studies	No. of Studies
Family Quality of Life	Caregiver Burden Index (CBI) [55]	Time Burden, Burden of Personal Development Limitations, Physical Burden, Social Burden, Emotional Burden	Ji et al. (2014) [43]	1
	Family Quality of Life (FQOL) [37]	Emotional Wellbeing, Family Wellbeing, Parenting Wellbeing, Physical/Material Wellbeing, Disability-Related Wellbeing	Hwang et al. (2015) [42]; Moody et al. (2019) [52]; Roberts et al. (2011) [54]	3
	Family Quality of Life Survey (FQOLS) [56]	About Your Family, Health of the Family, Support from Disability-Related Services, Leisure and Recreation, Community Interaction, Overall Family Quality of Life	Jones et al. (2017) [44]	1
	Par-DD-QoL (from Par-ENT-QoL) [57]	Emotional Score, Daily Disturbances Score, Global Score	Baghdadli et al. (2014) [10]	1
	Parenting Sense of Competence Scale (PSOC) [58]	Satisfaction, Interest, Efficacy	Mathew et al. (2019) [47]	1
	Parenting Stress Index (PSI) [38]	Parental Distress, Parent-Child Dysfunctional Interaction, Difficult Child	Paynter et al. (2018) [53]; Roberts et al. (2011) [54]	2
	WHOQOL-BREF [38]	Four Domains (Physical, Psychological, Social Relationships, Environment)	Derguy et al. (2018) [40]	1
	Quality of Life in Autism questionnaire (QoLA) [59]	Subscale A (Quality of Life), Subscale B (Impact of ASD Symptoms)	Due et al. (2018) [41]	1

### 3.3. Quality Assessment

We implemented the AXIS tool [34] to evaluate each of the 17 included papers. The average quality score was 13.18 out of a total of 20 points (min = 7, max = 19, *SD* = 3.68), indicating a moderate quality among studies, with two low, eight moderate, and seven high quality ratings (Table 3). Globally, the aims and the objectives were clearly indicated (question number one) and the design was appropriate for the stated aims (question number two). Only three papers [40,43,54], however, reported a specific calculation of the effect size (question number three) to justify the chosen sample. Since the selected interventions were specifically aimed at children with ASD and their parents, the target population was clearly defined in 12 studies (question number four), and the selection process was found to be adequate for most of the studies (questions number five and six). Non-responders were infrequent in the reviewed samples, nevertheless, only one study [43] explicitly reported the measures undertaken to address them (question number seven). The outcome variables were appropriately estimated with suitable instruments in approximately half of the included studies (question number eight, negative scores *n* = 7; question number nine, negative scores *n* = 7), and the statistical methods and data were globally specified in sufficient detail (questions from 10 to 12). Overall, the discussions and authors’ conclusions were justified by the achieved results, and the inherent limitations of the study designs were correctly presented (question number 18, negative scores *n* = 4).

### 3.4. Main Findings

#### 3.4.1. No Parental Involvement

In two studies, interventions were conducted entirely by professionals without any form of parental involvement [10,40]. Derguy et al. [40] found that access to child-focused psycho-educational intervention was positively associated with parental QoL. Baghdadli et al. [10] found that the number of hours of specialized intervention focused on externalizing difficulties of adolescents with ASD was negatively associated with parental QoL.

#### 3.4.2. Indirect Parental Involvement

Four studies used indirect strategies (e.g., parent training and support groups) to involve parents in the interventions [39,44,53,54]. In these cases, the main part of the intervention is centred on the child or the adolescent with ASD and is conducted in a clinical setting.

The results indicated an overall low level of QoL among parents who have been indirectly involved in an intervention for their child or adolescent with ASD. Specifically, Paynter et al. [53] found low levels of family adaptation (e.g., individual mental health, relationship quality, and family well-being) among fathers of children with ASD who participated to the centre-based early intervention. Interventions based on applied behaviour analysis (ABA) were associated with overall negative parent experiences because of the poor quality of the support received at home and the scarce coordination and guidance from the professionals [39]. In addition, findings by Jones et al. [44] showed that time on a waitlist for the ABA treatment was not associated with parental QoL. In contrast, Roberts et al.’s [54] randomised control trial revealed an increase in parent QoL after centre-based early interventions focused on functional impairments of children and on improving the parents’ knowledge of ASD.

#### 3.4.3. Direct Parental Involvement

Twelve studies used direct methods (e.g., parent-mediated home-based training, family-centred intervention, parent mentoring program) to involve parents in the interventions [41,42,43,45,47,48,49,50,51,52].

The majority of studies found that parental QoL improved after the intervention and, crucially, two studies found that parental direct involvement in the intervention was associated with greater parental QoL [41,43]. Specifically, parents of children with ASD showed significantly improved QoL after they participated in a parent-mediated communication-focused intervention [45,46], a family-centred intervention [49], an ABA-based home program [50], and the Colorado parent mentoring (CPM) program [52]. Moreover, parents of children with ASD who participated in a multidisciplinary parent education program reported increased mental, but not physical, QoL [43,48]. Consistently, Milbourn et al. [51] found that parents of children and adolescents with ASD reported that early access to intervention improved their QoL, especially in terms of fostering their involvement in their child’s daily life. However, three studies reported inconsistent results. Roberts et al. [48] found no significant changes in the QoL of parents of children with ASD after a home-based early intervention program. Similarly, Hwang et al. [42] found that mothers of children and adolescents with ASD did not report a significant increase in their QoL after they received a mindfulness parent-mediated home-based training. In addition, parents, especially mothers of children with ASD, who received an early intervention using the Early Start Denver Model showed lower parental well-being compared to the normative population [47].

## 4. Discussion

To our knowledge, this is the first review to examine the relationship between QoL and involvement in intervention among parents of children and adolescents with ASD. The 17 reviewed studies involved a total of 18 interventions targeting children and adolescents with ASD. Two studies reported no parental involvement in the intervention, three studies reported indirect parental involvement, and 11 studies reported direct parental involvement. One study [54] included both direct and indirect parental involvement in intervention.

The reviewed studies used heterogeneous designs and data collection methods and the majority of the studies had relatively small sample sizes [39,41,42,43,46,49,50,52,53,54], which limits our ability to draw firm conclusions about the association between parental QoL and involvement in interventions for youth with ASD.

From the limited data of the reviewed studies, the overall QoL of parents of children and adolescents with ASD appears to be shaped by the way they have been involved in the intervention. In line with a previous systematic review [24], our results showed that access to ASD services is a relevant protective factor not only for children, but also for parents [40]. Children with ASD can be expected to gain significant improvements when diagnosed early and when engaged in structured, intensive, evidence-based programs [61,62,63]. In contrast, difficulties in meeting the service needs of children with ASDs, ranging from general medical services to supportive services [64], can generate a vicious cycle of worsening symptoms and an overload of responsibility on parents who come to feel exhausted and ineffective [39]. However, the fact that one’s child with ASD receives treatment does not in itself guarantee an improvement in parental QoL. In fact, results showed that interventions based on the external difficulties of people with ASD, which do not involve parents in the intervention or involve them only indirectly, do not improve or even worsen parental QoL [10,39,44]. For example, interventions based on ABA are recognised as valid methods used for educating individuals with ASD [65,66], which are characterised by a strong asymmetry of roles of participants in the intervention. Qualified behaviour analysts design, develop, conduct, and directly oversee the ABA-programme that is focused on the child or the adolescent with ASD, while parents receive training to support their offspring in skill practice throughout the day. Behavioural child-focused interventions (e.g., ABA-based interventions), no matter how clinically effective, may be not sufficiently adapted to the broad family needs or may be unsuitable for a family situation. In contrast, when ABA-based interventions are implemented in a home setting and actively involved the whole family, parents report an overall better QoL [50]. In fact, interventions explicitly targeting children’s daily living skills in addition to the hallmark symptoms of ASD (i.e., social-communication deficits and repetitive, restrictive behaviours) may enhance parental QoL [54,67].

The majority of the studies examined in this review directly involved parents in the intervention. This may be a sign in the shift in perspective that is occurring in interventions for individuals with ASD and an increased awareness by professionals of the role of parents to implement effective interventions [46]. Parents who were directly involved in the intervention tended to report a higher QoL [43,45,46,49,50,51,52]. For example, in one study [43], 22 caregivers of children with ASD participated in an 8-week multidisciplinary education programme, with the intent to learn adaptive strategies and manage the problem behaviours of their child, and consequently to improve the intrafamilial QoL. Results indicated a significant improvement in family functioning, self-efficacy, and coping styles. Another study [49] provided post-diagnostic support to nearly 100 families and children, over a 12-month period. The project implemented a family-centred plan that showed improvements in children’s social and communication skills and overall stress reduction in parents. Yet another study [42] described an 8-week training program that comprised six mother–child dyads, aimed at teaching mindfulness strategies to better cope with problem behaviours of their children. At the end of the program, the participants reported positive effects in the targeted areas including parenting stress, intrafamilial QoL, and problematic behaviours. These results can be attributable to various reasons. When parents are actively involved by professionals, the intervention is more personalised and ecological [40]. This is also a way of empowering parents by recognising their expertise and contributions [68,69] and to develop constructive collaboration between different actors involved in the intervention [70]. Based on these findings, clinicians should assess and counsel parents, and identify potential barriers (e.g., lack of resolution of diagnosis) that may prevent them from actively participating in the intervention for their child or adolescent with ASD.

## 5. Limitations

The present systematic review comes with a number of limitations. Some depend on the relative novelty of the topic and on the absence of shared guidelines in the treatment of children and adolescents with ASD. The selected studies adopted a variety of methods and approaches (e.g., observational studies vs. randomised controlled trials), which limit the possibility to compare their findings. Another limitation regards our search and selection procedure. The construct of parental QoL is very broad and thus some studies may have evaluated some specific dimensions not included in our search keywords. Additionally, we only included articles published in English sources, which may have contributed to a selection bias by overlooking relevant articles published in other languages. In addition, we did not investigate the QoL of family as a whole (e.g., including siblings of children with ASD). However, parents are more generally actively involved in the care and intervention for their children. Furthermore, geographical coverage was dominated by Europe and Australia, therefore further research is needed in Asiatic cultural contexts [71]. Finally, the included studies recruited relatively small samples of self-selected participants who may not be necessarily representative of the general population.

## 6. Conclusions and Future Directions

Parental involvement in intervention for children or adolescents with ASD is a topic that is attracting increasing attention, as evidenced by the fact that parents were directly involved in interventions in most of the studies selected in this systematic review. The findings suggest that increasing parental involvement in the intervention for children or adolescents with ASD may be one way to promote their QoL. More precisely, this systematic review highlights that a constructive collaboration between professionals and parents in planning and executing interventions may promote more ecological results and better satisfaction among parents. This finding is consistent with the literature on parental QoL [24] and supports the relevance of an ecological and holistic approach in the research on QoL in parents of children with ASD [40]. This could help clinicians to better identify and address parental needs and enhance parenting resources that have a positive impact on the intervention.

Suggestions for future research put forward here include the use of longitudinal studies conducted with wider and demographically diverse samples that would include families from diverse geographical locations who have children with ASD of different ages (e.g., adults).

## Figures and Tables

**Table 3:** Quality assessments and total scores using the Appraisal Tool for Cross-Sectional Studies (AXIS).

Author (Year)	Q1	Q2	Q3	Q4	Q5	Q6	Q7	Q8	Q9	Q10	Q11	Q12	Q13 *	Q14	Q15	Q16	Q17	Q18	Q19 *	Q20	Quality Score/20	Quality Rating
Aandersson et al. (2017) [39]	Y	Y	N	N	Y	N	N	N	N	N	N	Y	N	N	Y	Y	Y	Y	N	Y	11	Moderate
Baghdadli et al. (2014) [10]	Y	N	N	Y	Y	Y	N	N	N	Y	N	Y	N	N	Y	Y	Y	Y	N	Y	13	Moderate
Derguy et al. (2018) [40]	Y	Y	Y	Y	Y	Y	N	Y	Y	Y	N	Y	N	N	Y	Y	Y	Y	N	Y	17	High
Due et al. (2018) [41]	Y	Y	N	N	Y	N	N	N	N	N	N	N	N	N	N	Y	Y	Y	N	Y	10	Moderate
Hwang et al. (2015) [42]	Y	Y	N	Y	Y	N	N	Y	Y	N	Y	Y	N	N	Y	Y	Y	Y	na	Y	14	Moderate
Ji et al. (2014) [43]	N	Y	Y	Y	Y	Y	Y	Y	Y	Y	Y	Y	N	Y	Y	Y	Y	Y	N	Y	19	High
Jones et al. (2017) [44]	Y	Y	N	Y	Y	Y	N	Y	Y	N	N	Y	N	N	Y	Y	Y	Y	N	Y	15	High
Leadbitter et al. (2018) [45]	Y	Y	N	N	Y	N	N	N	Y	N	N	Y	N	N	Y	Y	Y	N	N	Y	11	Moderate
Leadbitter et al. (2020) [46]	Y	Y	N	Y	Y	Y	N	Y	N	N	N	Y	N	N	Y	Y	Y	Y	N	Y	14	Moderate
Mathew et al. (2019) [47]	Y	Y	N	Y	Y	Y	N	Y	Y	Y	N	Y	N	N	Y	Y	Y	Y	N	Y	16	High
McConkey (2020) [48]	N	N	N	N	Y	Y	N	N	N	N	Y	Y	N	N	Y	Y	N	N	N	Y	9	Moderate
McConkey et al. (2020) [49]	N	Y	N	Y	N	N	N	N	N	N	N	Y	N	N	Y	N	N	N	N	Y	7	Low
McPhilemy & Dillenburger (2013) [50]	N	N	N	N	Y	N	N	N	N	N	N	Y	N	N	Y	Y	Y	N	na	Y	7	Low
Milbourn et al. (2017) [51]	Y	Y	N	Y	N	N	N	na	na	N	N	N	N	N	Y	Y	Y	Y	N	Y	10	Moderate
Moody et al. (2019) [52]	Y	Y	N	Y	Y	Y	N	Y	Y	Y	Y	Y	N	N	Y	Y	Y	Y	N	Y	17	High
Paynter et al. (2018) [53]	Y	Y	N	Y	Y	Y	na	Y	Y	N	Y	Y	N	N	Y	Y	Y	Y	N	Y	16	High
Roberts et al. (2011) [54]	Y	Y	Y	Y	Y	Y	N	Y	Y	Y	Y	Y	N	N	Y	Y	Y	Y	N	Y	18	High
**Mean**																					**13.18**	
**Standard Deviation**																					**3.68**	

Note: Yes = Y; No = N; Don’t know = na; Items 13 and 19 are score reversed (see [60]).

## Data Availability

Not applicable.

## Supplementary Materials

The following are available online at https://www.mdpi.com/article/10.3390/jpm11090894/s1, Table S1: Full-text articles excluded, with reasons (*n* = 79).
