# Supplementary material for: Parental Quality of Life and Involvement in Intervention for Children or Adolescents with Autism Spectrum Disorders: A Systematic Review
Source: J Pers Med. 2021 Sep 8;11(9):894. doi: 10.3390/jpm11090894 (PMC8469292; doi:10.3390/jpm11090894)
Supplement: Supplementary file 1 [file jpm-11-00894-s001.zip › jpm-1349615-supplementary.pdf]

Table S1

Full-text articles excluded, with reasons (n = 79)

REFERENCE	REASON FOR EXCLUSION
01. Alamri, D. A. F., Mahzari, Q. A., Shaqran, T., Albalawi, J. A., Alanazi, F. K., & Rheab. (2020). Impact of autism on parents/caregivers quality of life in Tabuk. *International Journal of Medical Research & Health Sciences*, *9*(1), 1–13.	No intervention
02. Al-Farsi, O. A., Al-Farsi, Y. M., Al-Sharbati, M. M., Al-Adawi, S., Cucchi, A., Essa, M. M., & Qoronfleh, M. W. (2020). Quality of life of caregivers of autism spectrum disorder, intellectual disability and typically developing children: A comparison study. *Applied Research in Quality of Life*.	No intervention
03. Arora, S., Goodall, S., Viney, R., & Einfeld, S. (2020). Health-related quality of life amongst primary caregivers of children with intellectual disability. *Journal of Intellectual Disability Research*, *64*(2), 103–116. https://doi.org/10.1111/jir.12701	Non-pertinent outcome
04. Avrech Bar, M., Shelef, L., & Bart, O. (2016). Do participation and self-efficacy of mothers to children with ASD predict their children's participation? *Research in Autism Spectrum Disorders*, *24*, 1–10. https://doi.org/10.1016/j.rasd.2016.01.002	No intervention
05. Bader, S. H., Barry, T. D., & Hann, J. A. H. (2015). The relation between parental expressed emotion and externalizing behaviors in children and adolescents with an autism spectrum disorder. *Focus on Autism and Other Developmental Disabilities*, *30*(1), 23–34. https://doi.org/10.1177/1088357614523065	No intervention
06. Benjak, T., Mavrinac, G. V., & Šimetin, I. P. (2009). Comparative study on self perceived health of parents of children with autism spectrum disorders and parents of nondisabled children in Croatia. In Mohammadi, MR (Ed.), *Croatian Medical Journal* (Vol. 50, Issue 4, pp. 403–409). https://doi.org/10.3325/cmj.2009.50.403	No intervention
07. Boehm, T. L., Carter, E. W., & Taylor, J. L. ound. (2015). Family quality of life during the transition to adulthood for individuals with intellectual disability and/or autism spectrum disorders. *American Journal on Intellectual and Developmental Disabilities*, *120*(5), 395–411.	Non-pertinent outcome
08. Bohadana, G., Morrissey, S., & Paynter, J. (2019). Self-compassion: A novel predictor of stress and quality of life in parents of children with autism spectrum disorder. *Journal of Autism and Developmental Disorders*, *49*(10), 4039–4052.	No intervention
09. Bravo-Benítez, J., Pérez-Marfil, M. N., Román-Alegre, B., & Cruz-Quintana, F. (2019). Grief experiences in family caregivers of children with autism spectrum disorder (ASD). *International Journal of Environmental Research and Public Health*, *16*(23). https://doi.org/10.3390/ijerph16234821	Non-pertinent outcome
10. Broady, T. R., Stoyles, G. J., & Morse, C. (2017). Understanding carers' lived experience of stigma: The voice of families with a child on the autism spectrum. *Health and Social Care in the Community*, *25*(1), 224–233. https://doi.org/10.1111/hsc.12297	Non-pertinent outcome
11. Cappe, É., Poirier, N., Sankey, C., Belzil, A., & Dionne, C. (2018). Quality of life of French Canadian parents raising a child with autism spectrum disorder and effects of psychosocial factors. *Quality of Life Research*, *27*(4), 955–967. https://doi.org/10.1007/s11136-017-1757-4	No intervention
12. Cappe, E., Wolff, M., Bobet, R., & Adrien, J. L. (2011). Quality of life: A key variable to consider in the evaluation of adjustment in parents of children with autism spectrum disorders and in the development of relevant support and assistance programmes. *Quality of Life Research*, *20*(8), 1279–1294. https://doi.org/10.1007/s11136-011-9861-3	Non-pertinent outcome
13. Chuang, I. C., Tseng, M. H., Lu, L., Shieh, J. Y., & Cermak, S. A. (2014). Predictors of the health-related quality of life in preschool children with autism spectrum disorders. *Research in Autism Spectrum Disorders*, *8*(9), 1062–1070. https://doi.org/10.1016/j.rasd.2014.05.015	No intervention
14. Craig, F., Operto, F. F., De Giacomo, A., Margari, L., Frolli, A., Conson, M., Ivagnes, S., Monaco, M., & Margari, F. (2016). Parenting stress among parents of children with neurodevelopmental disorders. *Psychiatry Research*, *242*, 121–129. https://doi.org/10.1016/j.psychres.2016.05.016	No intervention

REFERENCE	REASON FOR EXCLUSION
15. Ede, M. O., Anyanwu, J. I., Onuigbo, L. N., Ifelunni, C. O., Alabi-Oparaocha, F. C., Okenyi, E. C., Agu, M. A., Ugwuanyi, L. T., Ugwuanyi, C., Eseadi, C., Awoke, N. N., Nweze, T., & Victor-Aigbodion, V. (2020). Rational emotive family health therapy for reducing parenting stress in families of children with autism spectrum disorders: A group randomized control study. *Journal of Rational - Emotive and Cognitive - Behavior Therapy*, *38*(2), 243–271. https://doi.org/10.1007/s10942-020-00342-7	Non-pertinent outcome
16. Fisher, E. B. (2013). The impact of child's severity on quality-of-life among parents of children with autism spectrum disorder: The mediating role of optimism. *Journal of the Medical Association of Thailand = Chotmaihet Thangphaet*, *96*(10), 1313–1318.	No intervention
17. Frantz, R., Hansen, S. G., Squires, J., & Machalicek, W. (2018). Families as partners: Supporting family resiliency through early intervention. *Infants and Young Children*, *31*(1), 3–19. https://doi.org/10.1097/IYC.0000000000000109	Non pertinent
18. García-López, C., Sarriá, E., & Pozo, P. (2016). Parental self-efficacy and positive contributions regarding autism spectrum condition: An actor–partner interdependence model. *Journal of Autism and Developmental Disorders*, *46*(7), 2385–2398. https://doi.org/10.1007/s10803-016-2771-z	Non-pertinent outcome
19. Gardiner, E., & Iarocci, G. (2015). Family quality of life and asd: The role of child adaptive functioning and behavior problems. *Autism Research*, *8*(2), 199–213. https://doi.org/10.1002/aur.1442	No intervention
20. Garrido, D., Carballo, G., & Garcia-Retamero, R. (2020). Siblings of children with autism spectrum disorders: Social support and family quality of life. *Quality of Life Research*, *29*(5), 1193–1202. https://doi.org/10.1007/s11136-020-02429-1	No intervention
21. Giallo, R., Wood, C. E., Jellett, R., & Porter, R. (2013). Fatigue, wellbeing and parental self-efficacy in mothers of children with an autism spectrum disorder. *Autism*, *17*(4), 465–480. https://doi.org/10.1177/1362361311416830	No intervention
22. Giovagnoli, G., Postorino, V., Fatta, L. M., Sanges, V., De Peppo, L., Vassena, L., Rose, P. De, Vicari, S., & Mazzone, L. (2015). Behavioral and emotional profile and parental stress in preschool children with autism spectrum disorder. *Research in Developmental Disabilities*, *45–46*, 411–421. https://doi.org/10.1016/j.ridd.2015.08.006	No intervention
23. Goedeke, S., Shepherd, D., Landon, J., & Taylor, S. (2019). How perceived support relates to child autism symptoms and care-related stress in parents caring for a child with autism. *Research in Autism Spectrum Disorders*, *60*(January), 36–47. https://doi.org/10.1016/j.rasd.2019.01.005	No intervention
24. Gowda, G. S., Komal, S., Sanjay, T. N., Mishra, S., Kumar, C. N., & Math, S. B. (2019). Sociodemographic, legal, and clinical profiles of female forensic inpatients in Karnataka: A retrospective study. *Indian Journal of Psychological Medicine*, *41*(2), 138–143. https://doi.org/10.4103/IJPSYM.IJPSYM	Non pertinent
25. Harrop, C., McBee, M., & Boyd, B. A. (2016). How are child restricted and repetitive behaviors associated with caregiver stress over time? A parallel process multilevel growth model. *Journal of Autism and Developmental Disorders*, *46*(5), 1773–1783. https://doi.org/10.1007/s10803-016-2707-7	Non-pertinent outcome
26. Hou, Y. M., Stewart, L., Iao, L. S., & Wu, C. C. (2018). Parenting stress and depressive symptoms in Taiwanese mothers of young children with autism spectrum disorder: Association with children's behavioural problems. *Journal of Applied Research in Intellectual Disabilities*, *31*(6), 1113–1121. https://doi.org/10.1111/jar.12471	Non-pertinent outcome
27. Hsiao, Y. J. (2018). Autism spectrum disorders: Family demographics, parental stress, and family quality of life. *Journal of Policy and Practice in Intellectual Disabilities*, *15*(1), 70–79.	No intervention
28. Huang, Y. P., Chang, M. Y., Chi, Y. L., & Lai, F. C. (2014). Health-related quality of life in fathers of children with or without developmental disability: The mediating effect of parental stress. *Quality of Life Research*, *23*(1), 175–183.	No intervention
29. Iadarola, S., Levato, L., Harrison, B., Smith, T., Lecavalier, L., Johnson, C., Swiezy, N., Bearss, K., & Scahill, L. (2018). Teaching parents behavioral strategies for Autism Spectrum Disorder (ASD): Effects on stress, strain, and competence. *Journal of Autism and Developmental Disorders*, *48*(4), 1031–1040.	No intervention

REFERENCE	REASON FOR EXCLUSION
30. Ingersoll, B., & Berger, N. I. (2015). Parent engagement with a telehealth-based parent-mediated intervention program for children with autism spectrum disorders: Predictors of program use and parent outcomes. *Journal of Medical Internet Research*, *17*(10). https://doi.org/10.2196/jmir.4913	Non-pertinent outcome
31. Ji, B., Zhao, I., Turner, C., Sun, M., Yi, R., & Tang, S. (2014). Predictors of health-related quality of life in Chinese caregivers of children with autism spectrum disorders: A cross-sectional study. *Archives of Psychiatric Nursing*, *28*(5), 327–332. https://doi.org/10.1016/j.apnu.2014.06.001	No intervention
32. Kahana, E., Lee, J. E., Kahana, J., Goler, T., Kahana, B., Shick, S., Burk, E., & Barnes, K. (2015). Advancing the field. Childhood autism and proactive family coping: Intergenerational perspectives. *Journal of Intergenerational Relationships*, *13*(2), 150–166.	No empirical data
33. Kandeger, A., Guler, H. A., Egilmez, U., & Guler, O. (2018). Major depressive disorder comorbid severe hydrocephalus caused by Arnold – Chiari malformation Does exposure to a seclusion and restraint event during clerkship influence medical student' s attitudes toward psychiatry? *Indian Journal of Psychiatry*, *59*(4), 2017–2018. https://doi.org/10.4103/psychiatry.IndianJPsychiatry	Non pertinent
34. Karst, J. S., & van Hecke, A. V. (2012). Parent and family impact of autism spectrum disorders: A review and proposed model for intervention evaluation. *Clinical Child and Family Psychology Review*, *15*(3), 247–277.	No empirical data
35. Khanna, R., Madhavan, S. S., Smith, M. J., Patrick, J. H., Tworek, C., & Becker-Cottrill, B. (2011). Assessment of health-related quality of life among primary caregivers of children with autism spectrum disorders. *Journal of Autism and Developmental Disorders*, *41*(9), 1214–1227. https://doi.org/10.1007/s10803-010-1140-6	No intervention
36. Kousha, M., Attar, H. A., & Shoar, Z. (2016). Anxiety, depression, and quality of life in Iranian mothers of children with autism spectrum disorder. *Journal of Child Health Care*, *20*(3), 405–414. https://doi.org/10.1177/1367493515598644	No intervention
37. Kuhlthau, K., Payakachat, N., Delahaye, J., Hurson, J., Pyne, J. M., Kovacs, E., & Tilford, J. M. (2014). Quality of life for parents of children with autism spectrum disorders. *Research in Autism Spectrum Disorders*, *8*(10), 1339–1350.	No intervention
38. Kuru, N., & Piyal, B. (2018). Perceived social support and quality of life of parents of children with autism. *Nigerian Journal of Clinical Practice*, *21*(9), 1182–1189. https://doi.org/10.4103/njcp.njcp_13_18	No intervention
39. Lei, X., & Kantor, J. (2020). Social support and family quality of life in Chinese families of children with autism spectrum disorder: The mediating role of family cohesion and adaptability. *International Journal of Developmental Disabilities*. https://doi.org/10.1080/20473869.2020.1803706	No intervention
40. Lin, Y. N., Iao, L. S., Lee, Y. H., & Wu, C. C. (2020). Parenting stress and child behavior problems in young children with autism spectrum disorder: Transactional relations across time. *Journal of Autism and Developmental Disorders*.	Non-pertinent outcome
41. Lord, C., Brugha, T. S., Charman, T., Cusack, J., Dumas, G., Frazier, T., Jones, E. J. H., Jones, R. M., Pickles, A., State, M. W., Taylor, J. L., & Veenstra-VanderWeele, J. (2018). Autism spectrum disorder. *Nature Reviews Disease Primers*, *6*(1), 5. https://doi.org/10.1038/s41572-019-0138-4	No empirical data
42. Lovell, B., & Wetherell, M. A. (2020). Exploring the moderating role of benefit finding on the relationship between child problematic behaviours and psychological distress in caregivers of children with ASD. *Journal of Autism and Developmental Disorders*, *50*(2), 617–624. https://doi.org/10.1007/s10803-019-04300-w	No intervention
43. Lu, M. H., Wang, G. H., Lei, H., Shi, M. L., Zhu, R., & Jiang, F. (2018). Social support as mediator and moderator of the relationship between parenting stress and life satisfaction among the Chinese parents of children with ASD. *Journal of Autism and Developmental Disorders*, *48*(4), 1181–1188. https://doi.org/10.1007/s10803-017-3448-y	No intervention
44. Lu, M., Yang, G., Skora, E., Wang, G., Cai, Y., Sun, Q., & Li, W. (2015). Self-esteem, social support, and life satisfaction in Chinese parents of children with autism spectrum disorder. *Research in Autism Spectrum Disorders*, *17*, 70–77. https://doi.org/10.1016/j.rasd.2015.05.003	No intervention

REFERENCE	REASON FOR EXCLUSION
45. Lushin, V., & O'Brien, K. H. (2016). Parental Mental Health: Addressing the unmet needs of caregivers for children with autism spectrum disorders. *Journal of the American Academy of Child and Adolescent Psychiatry*, *55*(12), 1013–1015. https://doi.org/10.1016/j.jaac.2016.09.507	No empirical data
46. Markowitz, L. A., Reyes, C., Embacher, R. A., Speer, L. L., Roizen, N., & Frazier, T. W. (2016). Development and psychometric evaluation of a psychosocial quality-of-life questionnaire for individuals with autism and related developmental disorders. *Autism*, *20*(7), 832–844. https://doi.org/10.1177/1362361315611382	No intervention
47. Meadan, H., Halle, J. W., & Ebata, A. T. (2010). Families with children who have autism spectrum disorders: Stress and support. *Exceptional Children*, *77*(1), 7–36.	No empirical data
48. Mello, C., Rivard, M., Terroux, A., & Mercier, C. (2019). Quality of life in families of young children with autism spectrum disorder. *American Journal on Intellectual and Developmental Disabilities*, *124*(6), 535–548.	Non-pertinent outcome
49. Millau, M., Rivard, M., & Mello, C. (2019). Quality of life in immigrant parents of children with autism spectrum disorder: A comparison with parents from the host culture. *Journal of Child and Family Studies*, *28*(6), 1512–1523. https://doi.org/10.1007/s10826-019-01395-8	No intervention
50. Mugno, D., Ruta, L., D'Arrigo, V. G., & Mazzone, L. (2007). Impairment of quality of life in parents of children and adolescents with pervasive developmental disorder. *Health and Quality of Life Outcomes*, *5*. https://doi.org/10.1186/1477-7525-5-22	No intervention
51. Özgör, B. G., Aksu, H., & Eser, E. (2018). Factors affecting quality of life of caregivers of children diagnosed with autism spectrum disorder. *Indian Journal of Psychiatry*, *60*(3), 278–285. https://doi.org/10.4103/psychiatry.IndianJPsychiatry_300_17	No intervention
52. Plaza, J. C., Rico, G. M., & Grau-Sevilla, M. D. (2020). Coping strategies of fathers in early intervention services and parenting stress levels. *Journal of Psychological and Educational Research*, *28*(2), 119–145.	Non-pertinent outcome
53. Pozo, P., Sarriá, E., & Brioso, A. (2014). Family quality of life and psychological well-being in parents of children with autism spectrum disorders: A double ABCX model. *Journal of Intellectual Disability Research*, *58*(5), 442–458. https://doi.org/10.1111/jir.12042	No intervention
54. Pruitt, M. M., Willis, K., Timmons, L., & Ekas, N. V. (2016). The impact of maternal, child, and family characteristics on the daily well-being and parenting experiences of mothers of children with autism spectrum disorder. *Autism*, *20*(8), 973–985. https://doi.org/10.1177/1362361315620409	No intervention
55. Rayan, A., & Ahmad, M. (2016). Effectiveness of mindfulness-based interventions on quality of life and positive reappraisal coping among parents of children with autism spectrum disorder. *Research in Developmental Disabilities*, *55*, 185–196. https://doi.org/10.1016/j.ridd.2016.04.002	No empirical data
56. Reed, P., & Osborne, L. A. (2012). Diagnostic practice and its impacts on parental health and child behaviour problems in autism spectrum disorders. *Archives of Disease in Childhood*, *97*(10), 927–931.	Non-pertinent outcome
57. Reed, P., & Osborne, L. A. (2019). Reaction to diagnosis and subsequent health in mothers of children with autism spectrum disorder. *Autism*, *23*(6), 1442–1448. https://doi.org/10.1177/1362361318815641	Non-pertinent outcome
58. Rivard, M., Morin, M., Mercier, C., Terroux, A., Mello, C., & Lépine, A. (2017). Social validity of a training and coaching program for parents of children with autism spectrum disorder on a waiting list for early behavioral intervention. *Journal of Child and Family Studies*, *26*(3), 877–887. https://doi.org/10.1007/s10826-016-0604-5	Non-pertinent outcome
59. Robert, M., Leblanc, L., & Boyer, T. (2015). When satisfaction is not directly related to the support services received: Understanding parents' varied experiences with specialised services for children with developmental disabilities. *British Journal of Learning Disabilities*, *43*(3), 168–177. https://doi.org/10.1111/bld.12092	Non-pertinent outcome

REFERENCE	REASON FOR EXCLUSION
60. Rossetti, K. G., & Zlomke, K. R. (2021). Resourcefulness revisited: Further psychometric evaluation of resourcefulness scale. *Stress and Health*.	No empirical data
61. Russa, M. B., Matthews, A. L., & Owen-DeSchryver, J. S. (2015). Expanding supports to improve the lives of families of children with autism spectrum disorder. *Journal of Positive Behavior Interventions*, *17*(2), 95–104.	Non-pertinent outcome
62. Schertz, H. H., Lester, J. N., Erden, E., Safran, S., & Githens, P. (2020). Challenges and contributors to self-efficacy for caregivers of toddlers with autism. *Autism*, *24*(5), 1260–1272.	No intervention
63. Schlebusch, L., Dada, S., & Samuels, A. E. (2017). Family quality of life of South African families raising children with autism spectrum disorder. *Journal of Autism and Developmental Disorders*, *47*(7), 1966–1977. https://doi.org/10.1007/s10803-017-3102-8	No intervention
64. Selvakumar, N., & Panicker, A. (2020). Stress and coping styles in mothers of children with autism spectrum disorder. *Indian Journal of Psychological Medicine*, *42*(3), 225–232. https://doi.org/10.4103/IJPSYM.IJPSYM_333_19	Non-pertinent outcome
65. Shu, B. C., & Lung, F. W. (2005). The effect of support group on the mental health and quality of life for mothers with autistic children. *Journal of Intellectual Disability Research*, *49*(1), 47–53.	No intervention
66. Sim, A., Cordier, R., Vaz, S., Parsons, R., & Falkmer, T. (2017). Relationship satisfaction and dyadic coping in couples with a child with autism spectrum disorder. *Journal of Autism and Developmental Disorders*, *47*(11), 3562–3573. https://doi.org/10.1007/s10803-017-3275-1	No intervention
67. Srinivasaraghavan, R., Koshy, B., Devarajan, C., Beulah, R., & Glory, L. (2020). The impact of autism spectrum disorder in comparison with other neuro-developmental disorders in children on the family: Single centre experience. *Indian Journal of Psychological Medicine*, *42*(3), 233–237. https://doi.org/10.4103/IJPSYM.IJPSYM	No intervention
68. Stošić, J., Frey Škrinjar, J., & Preece, D. (2020). Families of children on the autism spectrum: Experience of daily life and impact of parent education. *Support for Learning*, *35*(2), 205–221. https://doi.org/10.1111/1467-9604.12300	No intervention
69. Tarver, J., Pearson, E., Edwards, G., Shirazi, A., Potter, L., Malhi, P., & Waite, J. (2020). Anxiety in autistic individuals who speak few or no words: A qualitative study of parental experience and anxiety management. *Autism*, *25*(2), 429–439. https://doi.org/10.1177/1362361320962366	Non-pertinent outcome
70. Taub, T., & Werner, S. (2016). What support resources contribute to family quality of life among religious and secular Jewish families of children with developmental disability? *Journal of Intellectual and Developmental Disability*, *41*(4), 348–359. https://doi.org/10.3109/13668250.2016.1228859	Non-pertinent outcome
71. Tehee, E., Honan, R., & Hevey, D. (2009). Factors contributing to stress in parents of individuals with autistic spectrum disorders. *Journal of Applied Research in Intellectual Disabilities*, *22*(1), 34–42.	Non-pertinent outcome
72. Tekola, B., Kinfe, M., Girma, F., Hanlon, C., & Hoekstra, R. A. (2020). Perceptions and experiences of stigma among parents of children with developmental disorders in Ethiopia: A qualitative study. *Social Science and Medicine*, *256*. https://doi.org/10.1016/j.socscimed.2020.113034	No intervention
73. Torbet, S., Proeve, M., & Roberts, R. M. (2019). Self-compassion: A protective factor for parents of children with autism spectrum disorder. *Mindfulness*, *10*(12), 2492–2506. https://doi.org/10.1007/s12671-019-01224-5	Non pertinent
74. Tümlü, C., & Akdoğan, R. (2019). Looking at life through a different window: Group counselling for the mothers of disabled children. *International Journal for the Advancement of Counselling*, *41*(2), 252–271. https://doi.org/10.1007/s10447-019-09373-x	No intervention

REFERENCE	REASON FOR EXCLUSION
75. Wakimizu, R., Yamaguchi, K., & Fujioka, H. (2017). Family empowerment and quality of life of parents raising children with developmental disabilities in 78 Japanese families. *International Journal of Nursing Sciences*, *4*(1), 38–45. https://doi.org/10.1016/j.ijnss.2016.12.004	No intervention
76. Walton, K. M. (2019). Leisure time and family functioning in families living with autism spectrum disorder. *Autism*, *23*(6), 1384–1397.	Non-pertinent outcome
77. Weitlauf, A. S., Broderick, N., Stainbrook, J. A., Taylor, J. L., Herrington, C. G., Nicholson, A. G., Santulli, M., Dykens, E. M., Juárez, A. P., & Warren, Z. E. (2020). Mindfulness-based stress reduction for parents implementing early intervention for autism: An RCT. *Pediatrics*, *145*, 81–92. https://doi.org/10.1542/peds.2019-1895K	No intervention
78. Weitlauf, A. S., Vehorn, A. C., Taylor, J. L., & Warren, Z. E. (2014). Relationship satisfaction, parenting stress, and depression in mothers of children with autism. *Autism*, *18*(2), 194–198. https://doi.org/10.1177/1362361312458039	No intervention
79. Wicks, R., Paynter, J., & Adams, D. (2019). Exploring the predictors of family outcomes of early intervention for children on the autism spectrum: An Australian cohort study. *Journal of Early Intervention*. https://doi.org/10.1177/1053815119883413	Non pertinent outcome
